# A Three-Hierarchy Evaluation of Polarimetric Performance of GF-3, Compared with ALOS-2/PALSAR-2 and RADARSAT-2

**DOI:** 10.3390/s19071493

**Published:** 2019-03-27

**Authors:** Zezhong Wang, Jian Jiao, Qiming Zeng, Junyi Liu

**Affiliations:** 1 Institute of Remote Sensing and Geographic Information System, School of Earth and Space Science, Peking University, Beijing 100871, China; zezhong_wang@pku.edu.com (Z.W.); jiaojian@pku.edu.cn (J.J.); 2 State Key Laboratory of Information Engineering in Surveying, Mapping and Remote Sensing, Wuhan University, Wuhan 430079, China; liujunyi_ljy@163.com

**Keywords:** GaoFen-3 (GF-3), polarimetric SAR (PolSAR), image quality, evaluation, calibration

## Abstract

GaoFen-3 (GF-3) is the first Chinese civilian multi-polarization synthetic aperture radar (SAR) satellite, launched on 10 August of 2016, and put into operation at the end of January 2017. The polarimetric SAR (PolSAR) system of GF-3 is able to provide quad-polarization (quad-pol) images in a variety of geophysical research and applications. However, this ability increases the complexity of maintaining image quality and calibration. As a result, to evaluate the quality of polarimetric data, polarimetric signatures are necessary to guarantee accuracy. Compared with some other operational space-borne PolSAR systems, such as ALOS-2/PALSAR-2 (ALOS-2) and RADARSAT-2, GF-3 has less reported calibration and image quality files, forcing users to validate the quality of polarimetric imagery of GF-3 before quantitative applications. In this study, without the validation data obtained from a calibration infrastructure, an innovative, three-hierarchy strategy was proposed to assess PolSAR data quality, in which the performance of GF-3 data was evaluated with ALOS-2 and RADARSAT-2 data as references. Experimental results suggested that: (1) PolSAR data of GF-3 satisfied backscatter reciprocity, similar with that of RADARSAT-2; (2) most of the GF-3 PolSAR images had no signs of polarimetric distortion affecting decomposition, and the system of GF-3 may have been improved around May 2017; and (3) the classification accuracy of GF-3 varied from 75.0% to 91.4% because of changing image-acquiring situations. In conclusion, the proposed three-hierarchy approach has the ability to evaluate polarimetric performance. It proved that the residual polarimetric distortion of calibrated GF-3 PolSAR data remained at an insignificant level, with reference to that of ALOS-2 and RADARSAT-2, and imposed no significant impact on the polarimetric decomposition components and classification accuracy.

## 1. Introduction

GaoFen-3 (GF-3) was launched on 10 August of 2016 and was put into operation at the end of January, 2017 [1]. It is the first Chinese space-borne multi-polarization co-/cross-imaging radar mission in C-band, with a fully polarimetric quad-polarization (quad-pol) mode [2]. The quad-pol mode provides data with at least 40 beams and ground range resolutions of about 8 m and 25 m [3]. These polarimetric data are expected to be substantially applied in sea and ocean monitoring, disaster reduction, water conservancy, and meteorology [4]. The performance of these applications depends extremely on the polarimetric fidelity. This arouses special concern in users of GF-3 polarimetric data about the operations of polarimetric calibration and quality of polarimetric signatures. Despite the introduction of the synthetic aperture radar (SAR) payload design and the report of in-orbit tests and evaluations [2,4], these users still have to validate the quality of data before quantitative applications because of the lack of periodic updated calibration files. Meanwhile, in contrast to the PolSAR system of GF-3, another two space-borne polarimetric SAR (PolSAR) systems, ALOS-2/PALSAR-2 (ALOS-2) and RADARSAT-2, both have the sophisticated images quality subsystem (IQS) [5,6]. In addition, infrastructure and procedures designed to support the image quality and calibration operations, substantial and sustainable updates of calibration files, and annual status of the mission have been reported in both systems [5,6,7,8,9,10,11,12,13,14,15,16,17]. Hence, it is essential to evaluate the quality of GF-3 polarimetric data and to achieve the similar quality compared with ALOS-2 and RADARSAT-2.

Quality assessment of polarimetric data refers to the estimation of the transmission distortion and reception distortion, each as a 2 × 2 matrix containing channel imbalances (CIs) and crosstalk terms (CTs), where polarimetric calibration is used to compensate the distortions [7,14]. In the campaign of the PolSAR mission, quality assessment and polarimetric calibration are always performed together. In terms of the GF-3 mission, through trihedral corner reflectors (TCRs) and grassland images in the Etuoke Banner of Inner Mongolia, China, quality assessment and polarimetric calibration was conducted by Chang et al. [18]. However, without the validation data derived from calibration infrastructure, users of GF-3 PolSAR data have to develop more strategies for the evaluation of polarimetric performance. Taking calibrated PolSAR images of RADARSAT-2 as reference, Jiang et al. found special natural objects and then selected the measured polarimetric signals of those objects to estimate CIs and CTs [3]. Nevertheless, in contrast to evaluation of polarimetric fidelity by direct means, this study proposed an indirect, three-hierarchy strategy to assess the data quality of GF-3 based on images themselves and its application with ALOS-2 and RADARSAT-2 as references. The three-hierarchy evaluation starts from an image histogram, to a polarimetric decomposition result, and ends at an image classification result. The histogram of polarimetric signals presents a statistical characteristic of each polarimetric channel. Further, results of polarimetric decomposition and classification provide an indirect indication of polarimetric fidelity. This originates from the impacts CTs and CIs have effect on polarimetric decomposition and classification [19]. These impacts highlight that CTs lead to a decrease of polarimetric entropy and an increase of volume scattering components; CIs bring about deflection of the alpha parameter of eigenvalue-based decomposition and enlarging of the model-based decomposition error; and both CIs and CTs play a negative role in classification accuracy.

## 2. Methodology

**Figure 1:**
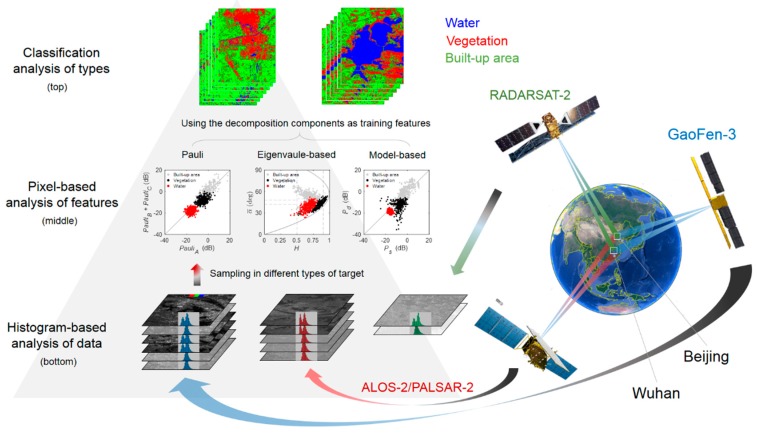
A three-hierarchy framework to evaluate the quality of polarimetric SAR data.

The proposed three-hierarchy evaluation framework (Figure 1) consisted of a histogram-based analysis, pixel-based analysis, and classification assessment. The histogram-based analysis involved two hypotheses: (1) PolSAR images satisfy backscatter reciprocities ($S_{hv}=S_{vh}$) [7], and the intensity differences between HV and VH should reach zero for most of the pixels if the data has insignificant polarimetric distortion; (2) PolSAR images with similar Equivalent Number of Looks (ENL) should have similar statistical distributions in the same area [20], and the polarimetric distortions of the data of GF-3 and other sensors are in a similar level if those data have similar histograms. The pixel-based analysis was under the hypothesis that polarimetric distortion enlarged the polarimetric decomposition error [19,21], and the polarimetric distortion was considered insignificant when the polarimetric decomposition results of different types of samples presented specific backscattering features and significant separation. The hypothesis of the classification assessment assumed that polarimetric distortion decreases the classification accuracy [22], and considering the impact of images acquiring situation, such as operational band, incidence angle and resolution, a higher classification accuracy indicated better polarimetric fidelity and image quality. Overall, the three-hierarchy framework starts at the bottom of the data, continues through the middle of the polarimetric decomposition feature, and ends at the top of the application.

### 2.1. Histogram-Based Analysis

A histogram helps to clearly present the overall distribution of the digital image. In the present study, Bhattacharyya distance (*Bd*) was used for quantitative measurement of the similarities of the histogram [23]. In this paper, Bhattacharyya distance was selected for its simplicity and effectiveness in quantitative comparisons of images.

Bhattacharyya distance is defined as:

(1) $$Bd\left(H_{1},H_{2}\right)=-\ln\left(\overset{n}{\underset{i=0}{\sum }}\sqrt{H_{1}\left(i\right)H_{2}\left(i\right)}\right),$$

where *H_1_* and *H_2_* are the frequency of normalized histograms, and *n* is the number of bins in the histogram. The range of *Bd* was [0−+∞) and the same images presented the minimum value of 0.

### 2.2. Pixel-Based Analysis

The scattering matrix of each pixel in a PolSAR image contained full polarimetric information of the corresponding target, which can be used to detect and distinguish land-cover types. Target decomposition theory, making use of a scattering matrix or its second-order statistics, has been widely applied in expressing the scattering mechanisms that lead to the polarimetric signatures seen in a PolSAR image, such as surface scattering, double-bounce scattering, and volume scattering [24,25]. Based on target decomposition theory, this study obtained the scattering mechanism of sampling pixels performed in a PolSAR image and analyzed whether that appropriately reflected the backscatter property of the corresponding land-cover types. Two theories of target decomposition were categorized: coherent target decomposition (CTD) and incoherent target decomposition (ITD). CTD dealt with decomposition of the scattering matrix, whereas ITD made use of the second-order statistics such as coherency or the covariance matrix [26]. In ITD, two representative groups were distinguished: eigenvalue-based approaches (E-ITD) and mode-based approaches (M-ITD). One representative theory in CTD, E-ITD, and M-ITD, respectively, was selected here.

#### 2.2.1. Coherent Decomposition

Pauli decomposition, as one of the most known and applied CTD theories, decomposes a scattering matrix using a Pauli matrix where every base matrix is associated to a basic scattering mechanism [24]. This model is expressed as:

(2) $$S=\left[\begin{array}{cc} S_{hh} & S_{hv}\\ S_{vh} & S_{vv} \end{array}\right]=A\frac{1}{\sqrt{2}}\left[\begin{array}{cc} 1 & 0\\ 0 & 1 \end{array}\right]+B\frac{1}{\sqrt{2}}\left[\begin{array}{cc} 1 & 0\\ 0 & -1 \end{array}\right]+C\frac{1}{\sqrt{2}}\left[\begin{array}{cc} 0 & 1\\ 1 & 0 \end{array}\right]+D\frac{1}{\sqrt{2}}\left[\begin{array}{cc} 0 & -j\\ j & 0 \end{array}\right],$$

where *S* is the 2 × 2 Sinclair matrix, and *S_hv_* is the scattering coefficient of horizontal transmitting (*h*) and vertical receiving polarization (*v*), and the other three coefficients are defined similarly. Pauli decomposition can be interpreted as the coherent decomposition of the Sinclair matrix into four physical mechanisms: (A) surface scattering, (B) double-bounce scattering of orthogonal dihedral corners, (C) cross-polarization components, and (D) all asymmetric components. Among four components for the Pauli decomposition result, only the first three components were chosen for further analysis because the fourth component was not associated with any specific physical scattering mechanism.

#### 2.2.2. Eigenvalue-Based Decomposition

For development of target decomposition approaches based on the Huynen theory, there were three different forms of decomposition result, as there were three completely different matrixes with the first rank corresponding to the coherency matrix [27]. To obtain a unique form of the decomposition result, Cloude first proposed the eigenvalue-based decomposition method because of the invariability of eigenvalues, regardless of the change of base [28]. Further, three parameters (*H,* $\bar{\alpha }$ and *A*) related to the eigenvalues and eigenvectors of the coherence matrix were developed to enterprise the scattering mechanisms of the target [29]. Then, *H/*$\bar{\alpha }$*/A* decomposition theory with wide applications was used in this study. In this theory, scattering entropy (*H*) describes the randomness of eigenvalues of the coherency matrix, mean alpha angle ($\bar{\alpha }$) is one of the mean parameters of the dominant scattering mechanism from the coherency matrix, and anisotropy (*A*) presents the relationship between the second and the third eigenvalues, as the entropy (*H*) does not completely describe the ratio of the eigenvalues [29]. *H,* $\bar{\alpha }$ and *A* were calculated as:

(3) $$H=\overset{3}{\underset{i=1}{\sum }}\left(-P_{i}\log_{3}P_{i}\right),P_{i}=\frac{\lambda_{i}}{\lambda_{1}+\lambda_{2}+\lambda_{3}},$$

(4) $$\bar{\alpha }=\overset{3}{\underset{i=1}{\sum }}P_{i}\lambda_{i},$$

(5) $$A=\frac{\lambda_{2}-\lambda_{3}}{\lambda_{2}+\lambda_{3}},$$

where *λ_i_* is the eigenvalue of the coherency matrix, and *P_i_* is the discrete probability distribution of the eigenvalues. The coherency matrix was the second-order statistical matrix acquired from the Sinclair matrix. For reciprocal backscatter SAR, this could be obtained by:

(6) $$k=\frac{1}{\sqrt{2}}{\left[\begin{array}{ccc} S_{hh}+S_{vv} & S_{hh}-S_{vv} & 2S_{hv} \end{array}\right]}^{T},$$

(7) $$T_{3}=k\cdot k^{*T}=\left[\begin{array}{ccc} T_{11} & T_{12} & T_{13}\\ T_{21} & T_{22} & T_{23}\\ T_{31} & T_{32} & T_{33} \end{array}\right],$$

where *T_3_* is the coherency matrix used for ITD decomposition.

#### 2.2.3. Model-Based Decomposition

Before the model-based decomposition theory was proposed, the existing decompositions were focused so much on mathematics that they could not be easily interpreted as physical scattering mechanisms [25]. Then, based on the physical model of some simple scattering mechanisms, many decomposition methods were developed to depict the backscattering properties of the target. The well-known four-component decomposition method was put forward by Yamaguchi et al., based on four physical models [30]. It was selected here because of its good performance in depicting the basic physical scattering mechanism of targets in urban areas. The four-component decomposition model is expressed as:

(8) $$T_{3}=P_{s}T_{\mathrm{surface}}+P_{d}T_{\mathrm{double}}+P_{v}T_{\mathrm{volume}}+P_{c}T_{\mathrm{helix}}$$

where ***T**_3_* is the coherency matrix given in (7). The four scattering components represent the surface scatter (*P_s_*), double-bounce scatter (*P_d_*), volume scatter (*P_v_*), and helix scatter (*P_c_*), respectively.

### 2.3. Land-Cover Classification

In this study, pixels in PolSAR images were categorized into three land-cover types, including built-up areas, vegetation, and water. In this paper, a support vector machine (SVM) was used as the classifier for its supervised process and better performance in classification accuracy compared with other popular classifiers, such as maximum likelihood and k-nearest neighbor [31,32,33]. The SVM algorithm is a binary, linear classifier that uses a set of training samples, each of which is marked as belonging to one or the other of two categories, to build a model that assigns each pixel to one category or the other [32]. To keep consistent with process in pixel-based analyses, ten decomposition components obtained by Pauli decomposition, eigenvalue-based decomposition, and model-based decomposition were used as the training features in the SVM classifier.

## 3. Experiments

### 3.1. Study Area and Data

#### 3.1.1. Study Areas

**Figure 2:**
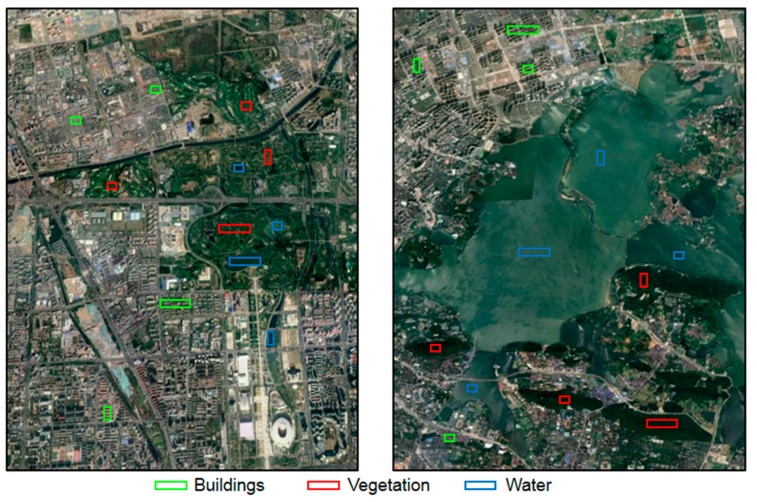
Google Earth images of the study areas in Beijing (left) and in Wuhan (right).

Current, area-wide information management in highly dynamic urban settings is critically required for future development. In this regard, PolSAR data offered the possibility of a fast and area-wide assessment of urban changes and developments. Hence, two study areas in Beijing and Wuhan, with rapid economic growth and urbanization in recent years, were selected to evaluate the performance of GF-3 PolSAR data. Beijing, the capital of China, is located in the North China Plain between latitudes 39°59′ and 40°04′ N and longitudes 116°21′ E and 116°25′ E; Wuhan, the capital of Hunan Province, is located in the Jianghan Plain, central China, between latitudes 30°31′ and 30°36′ N and longitudes 114°22′ E and 114°26′ E (Figure 2).

Although both Beijing and Wuhan are metropolises of China with large populations, there are some differences between them. Combining both traditional and modern architecture, Beijing is one of the oldest cities in the world, with many historical sites. However, Wuhan is a typical modern city with huge development in the past ten years. In addition, water area in Beijing is distinctly less than that in Wuhan, since Wuhan is developed along the Changjiang (Yangtze) River. As shown in Figure 2, the study areas of Beijing consisted of a large proportion of built-up areas and vegetation, but only a small proportion of water. By contrast, almost half of the study area in Wuhan was water; the rest was built-up areas and vegetation. For the histogram-based experiments, all the PolSAR images over Beijing were clipped into the subset as the left map in Figure 2, and all the PolSAR images over Wuhan were clipped into the subset as the right map in Figure 2. Further, the samples for pixel-based experiments and for training the classifier were selected over the region, demarcated by boxes with green, red, and blue lines.

#### 3.1.2. Polarimetric SAR Data and Ground Reference Data

Many factors have impacts on the polarimetric performance of SAR imaging, including sensor parameters and image acquisition situations. To make a general cross-comparison, we made efforts to collect more PolSAR data from GF-3, ALOS-2, and RADARSAT-2 in the study areas as much as possible. A total of 13 PolSAR images in two areas were collected, including three of GF-3, three of ALOS-2, and one of RADARSAT-2 in Beijing, and three of GF-3, two of ALOS-2, and one of RADARSAT-2 in Wuhan. The 13 images had variable parameters, such as incidence angles and imaging times (Table 1). The nominal resolutions of GF-3, RADARSAT-2, and ALOS-2 were similar, at about 8 m. Although the operating band of GF-3 (C-band) was different from ALOS-2 (L-band), we conducted a comparison between them. The first reason was that the comparison of GF-3 with ALOS-2 was under the hypothesis that if the distortion effect was insignificant, the GF-3 (C-band) should present more surface scattering phenomena and less double-bounce phenomena than ALOS-2 (L-band) in forest areas with a dense canopy. Another reason was that over each study site, we collected much more GF-3 and ALOS-2 data than RADARSAT-2 data. Thus, the data quantity of GF-3 and ALOS-2 made it possible to compare the stability of these sensors, to some extent.

Ground truth data were collected through fieldwork from 2017 to 2018. Ground reference data were used to select samples for pixel-based analysis. Also, a total of 80% of the data were randomly selected to train the classifier, and the remaining 20% were used to validate classification accuracy. The locations of selected samples in optical images of Google Earth are presented in Figure 2. The samples of built-up areas included residential areas, commercial buildings, grounds, and roads. The building structures in the sample areas of Beijing and Wuhan were not completely the same. Vegetation samples in Beijing were mostly trees in forest parks, and the remaining parts were grasslands. In Wuhan, by contrast, all the vegetation samples were selected in mountain forests. In regards to water, the samples were selected in artificial lakes over Beijing but in natural lakes over Wuhan.

#### 3.1.3. Image Processing

In data pre-processing, radiometric calibrations of GF-3, ALOS-2, and RADARSAT-2 were carried out using algorithms developed by the China Academy of Space Technology (CAST), the Japan Aerospace Exploration Agency (JAXA), and the Canadian Space Agency (CSA), respectively [2,16,34].

The GF-3 digital image was calibrated as:

(9) $$\sigma^{o}{}_{slc}=10\log_{10}\left[\left(I^{2}+Q^{2}\right)\cdot {\left(\frac{Q_{v}}{32767}\right)}^{2}\right]-K_{dB},$$

where *σ^o^_slc_* is the backscattering coefficient (dB); *I* and *Q* are the real and imagery parts of the complex image, respectively; *Q_v_* is the maximum value before image quantization; and *K_d_*_B_ is the calibration constant. *Q_v_* and *K_dB_* are both supplied in the header file.

The ALOS-2 digital image was calculated as:

(10) $$\sigma^{o}{}_{slc}=10\log_{10}\left[\left(I^{2}+Q^{2}\right)\right]+CF_{1}-A,$$

where *CF_1_* and *A* are the calibration coefficients. *CF_1_* for PALSAR-2 JAXA standard product was obtained as −83 dB. *A* for PALSAR-2 JAXA standard SLC data was equal to −32 dB [16].

The RADARSAT-2 digital image was calibrated as:

(11) $$\sigma^{o}{}_{slc}=10\log_{10}\left[\frac{\left(I^{2}+Q^{2}\right)+B}{A}\right],$$

where *B* and *A* are the offset and the gain, respectively, both supplied in the LUT (look-up-table) file [34]. As to the pixel-based analysis and classification, all using PolSAR images were processed with a 5 × 3 multilook (azimuth × range), and were georeferenced using the WGS84 reference ellipsoid.

### 3.2. Results

#### 3.2.1. Backscatter Reciprocity

**Figure 3:**
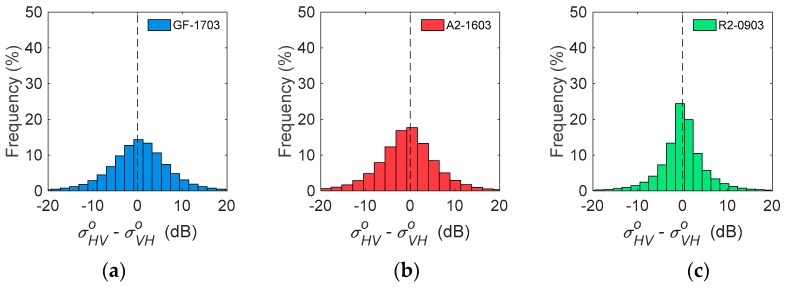
Histograms of the difference between $\sigma_{HV}^{o}$ and $\sigma_{VH}^{o}$ for GF-1703 (**a**), A2-1603 (**b**), and R2-0903 (**c**) over Beijing.

To evaluate the backscatter reciprocity ($S_{hv}=S_{vh}$) of PolSAR images, three images with the same acquiring season were selected. The difference of backscatter coefficients between HV and VH of GF-1703, A2-1603, and R2-0903 was computed, and their histograms are presented in Figure 3.$\sigma_{HV}^{o}$ was sigma0 (backscattering coefficient) of horizontal transmitting (*h*) and vertical receiving polarization (*v*), and the other three coefficients ($\sigma_{HH}^{o}$, $\sigma_{VV}^{o}$ and $\sigma_{VH}^{o}$) were defined similarly. Then, $\sigma_{HV}^{o}-\sigma_{VH}^{o}$ represented the difference between HV and VH used to evaluate backscatter reciprocity. The histogram of GF-1703 showed that most of the pixels were concentrated on zero, similar with A2-1603 (*Bd* = 0.01) and R2-0903 (*Bd* = 0.02). As indicated by the statistical results, GF-3 had similar percentages of pixels, with $\sigma_{HV}^{o}-\sigma_{VH}^{o}$ lower than any specific values as ALOS-2, e.g., 1 dB, 2 dB, 3 dB, 5 dB, and 10 dB, and the percentages were lower than that of RADARSAT-2 (Table 2).

#### 3.2.2. Distribution of Backscattering Coefficients

**Figure 4:**
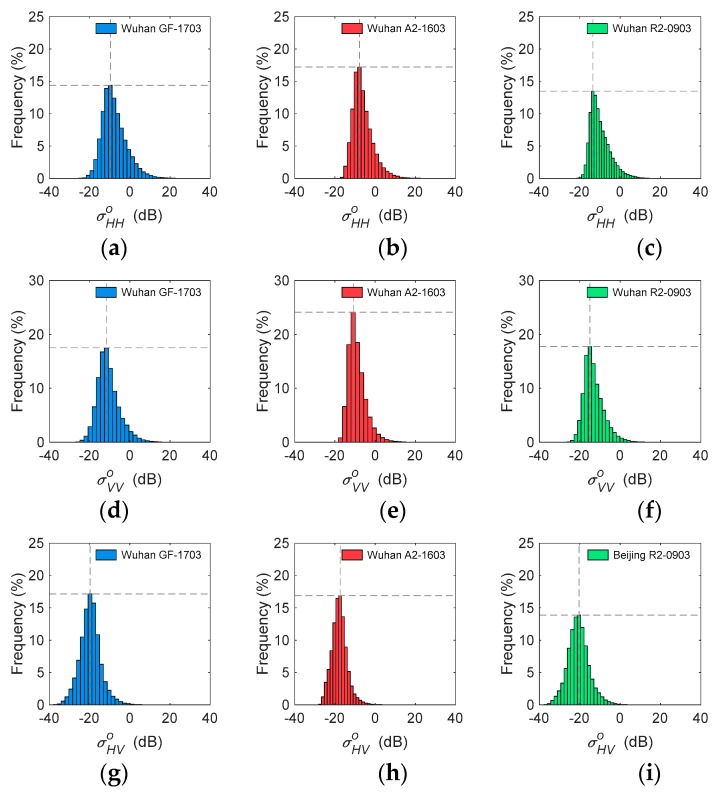
Histograms of GF-1703, A2-1603, and R2-0903 of $\sigma_{HH}^{o}$ (**a**–**c**), $\sigma_{VV}^{o}$ (**d**–**f**), and $\sigma_{HV}^{o}$ (**g**–**i**) in Beijing.

Considering the impact of season and incidence angle, one group of images (GF-1703, A2-1603, and R2-0903) obtained in the same season were selected over Beijing, and another group of images (GF-1702, A2-1601, and R2-1607) with a similar incidence angle around 37° were selected over Wuhan to compare the distribution of backscatter coefficients. As shown in Figure 4, GF-1703 presented more similar characteristics with R2-0903 because it operated at the same frequency as C-band compared to A2-1603. Histograms of $\sigma_{HH}^{o}$ showed that GF-1703 and R2-0903 had the same highest frequency (14%), but A2-1603 had the highest frequency at 17%. As to the histograms of $\sigma_{VV}^{o}$, GF-1703 and R2-0903 also displayed the same highest frequencies (18%), but A2-1603 reached the highest frequency of 24%. In addition, both GF-1703 and R2-0903 had the highest frequency at −20 dB $\sigma_{HV}^{o}$, but A2-1603 reached the highest frequency at −17 dB $\sigma_{HV}^{o}$. The similarity analysis verified the observation of histograms, with the Bhattacharyya distances between GF-3 and RADARSAT-2 presenting significantly lower values than those between GF-3 and ALOS-2 for all $\sigma_{HH}^{o}$, $\sigma_{VV}^{o}$ and $\sigma_{HV}^{o}$. In particular, GF-1710 and R2-0903 obtained high similarities of $\sigma_{HH}^{o}$ and $\sigma_{VV}^{o}$ (*Bd* < 0.05). This was the expected performance for GF-3, compared with RADARSAT-2 operating at C-band and ALOS-2 operating at L-band (Table 3).

**Figure 5:**
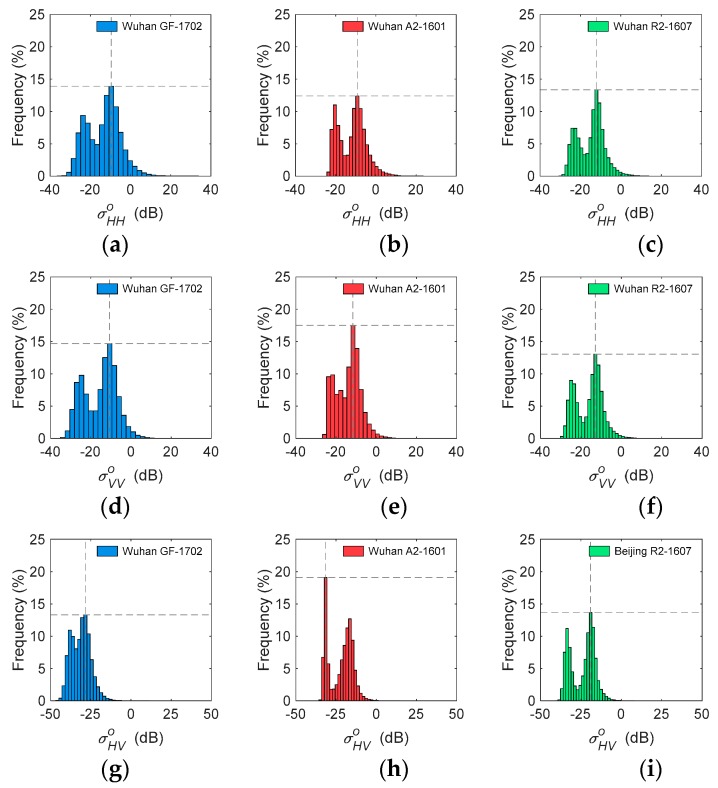
Histograms of GF-1702, A2-1601, and R2-1607 of $\sigma_{HH}^{o}$ (**a**–**c**), $\sigma_{VV}^{o}$ (**d**–**f**), and $\sigma_{HV}^{o}$ (**g**–**i**) in Wuhan.

In Wuhan, GF-1702, A2-1601, and R2-1607 were selected for their similar incidence angles, but they were obtained in different seasons. Notably, all histograms showed double peaks (Figure 5) because there were large parts of water in the study area. The Bhattacharyya distances between GF-1702, A2-1601, and R2-16037 ranged from 0.10 to 0.69, and were generally larger than that in Beijing. GF-1702 and A2-1601 reached a high similarity in $\sigma_{HH}^{o}$ (*Bd* = 0.1); while GF-1710 and R2-0903 had a medium similarity in $\sigma_{VV}^{o}$, with a *Bd* around 0.5. This meant that the observed backscattering coefficients did not only depend on frequency, but were impacted by the season. The histogram-based analysis indicated that GF-3 had a similar histogram with the data of other sensors when they had the same operating band and image-obtaining season.

#### 3.2.3. Polarimetric Performance of Target

**Figure 6:**
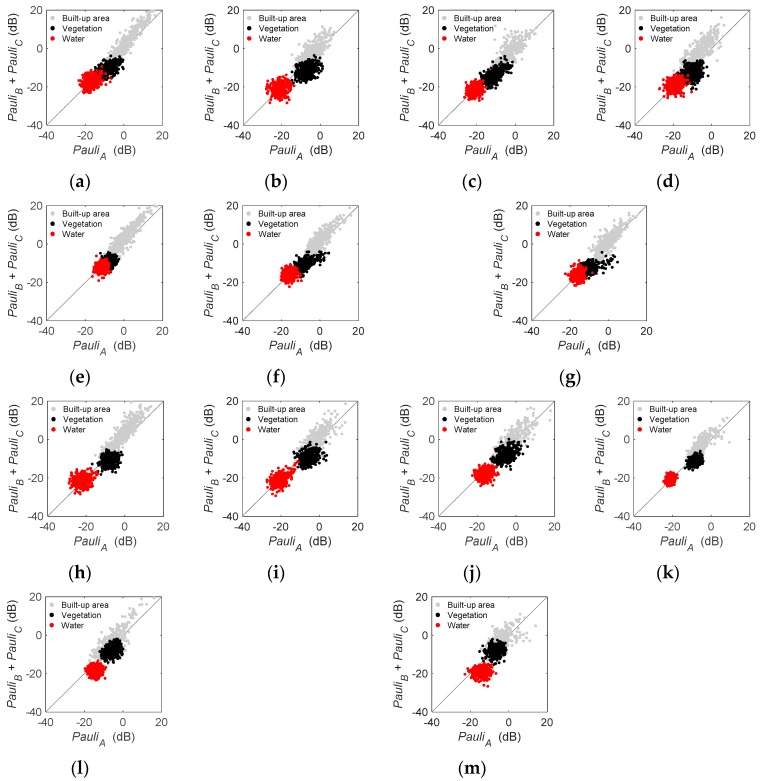
Scatter diagrams of built-up areas, vegetation, and water in Pauli-decomposed powers. (**a**) GF-1703, (**b**) GF-1710, (**c**) GF-1712, (**d**) R2-0903, (**e**) A2-1603, (**f**) A2-1610, (**g**) A2-1612, (**h**) GF-1702, (**i**) GF-1705, (**j**) GF-1708, (**k**) R2-1607, (**l**) A2-1504, and (**m**) A2-1601.

In this paper, we assumed the first component (*Pauli_A_*) of the Pauli decomposition represented surface scattering power, and that the sum (*Pauli_B_ + Pauli_C_*) of the second and third components represented compound scattering based on dihedral structures and dipoles [25,30]. The physical meaning of *Pauli_B_ + Pauli_C_* corresponded to the scatterers. When HH was superior to VV in the radar of built-up areas, the main contribution of *Pauli_B_* was made by orthogonal ground–wall structures, and the main source of the cross-pol component (*Pauli_C_*) was the radar from rotated dihedral structures. Thus, *Pauli_B_ + Pauli_C_* could be seen as the double-bounce scattering power of the compound [35,36]. When HH and VV were almost equal in the radar of forest canopy, the contribution of double-bounce scattering (*Pauli_B_*) was small, and the main source of *Pauli_B_ + Pauli_C_* could be seen as volume scattering from dipoles [36]. When the backscattering intensities were low over water, *Pauli_B_ + Pauli_C_* had little physical meaning under the noise effect. It was also noticed that water and vegetation were always recognized as distributed targets (or incoherent targets), and over those areas it was difficult to give a practical, physically-based interpretation of the components of the Pauli decomposition. Since the targets in urban areas seem to be coherent with slight speckle noise, the results of the Pauli decomposition in urban areas presented expected double-bounce scattering phenomena for all the three sensors with higher *Pauli_B_ + Pauli_C_* values than *Pauli_A_*. It was interesting that many dots in Figure 6 were aligned with the curve *x* = *y* for all the three sensors. This could be explained that most of the selected samples were incoherent targets so that the components acquired from coherent decomposition (Pauli decomposition) may be insufficient to enterprise the scattering mechanism, i.e., *Pauli_A_* and *Pauli_B_ + Pauli_C_* had little physical meaning. For example, in water and vegetation areas, most samples were incoherent targets, and they obtained almost equal values in both *Pauli_B_ + Pauli_C_* and *Pauli_A_*. However, built-up areas, vegetation, and water were clearly discriminated by the intensity of *Pauli_A_* and *Pauli_B_ + Pauli_C_*. As shown in Figure 6, both *Pauli_A_* and *Pauli_B_ + Pauli_C_* showed much higher values for built-up areas, medium values for vegetation, and much lower values for water.

**Figure 7:**
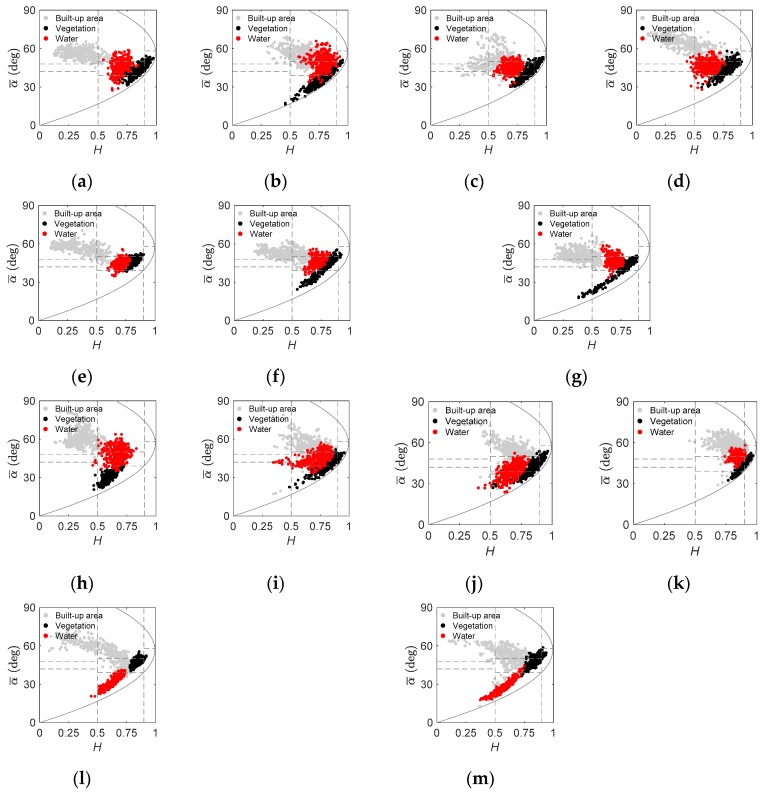
H-$\bar{\alpha }$ distribution of built-up areas, vegetation, and water. (**a**) GF-1703, (**b**) GF-1710, (**c**) GF-1712, (**d**) R2-0903, (**e**) A2-1603, (**f**) A2-1610, (**g**) A2-1612, (**h**) GF-1702, (**i**) GF-1705, (**j**) GF-1708, (**k**) R2-1607, (**l**) A2-1504, and (**m**) A2-1601.

As shown from Figure 7, variable H/$\bar{\alpha }$*/A* decomposition results of the 13 images were obtained. For built-up areas, most of the pixels in GF-1703, GF-1712, and GF-1702 were located in the upper left-hand portion of the *H*–$\bar{\alpha }$ map, similar with those of A2-1603, A2-1610, A2-1612, and R2-0903, which were likely provided by isolated dihedral scatterers. By contrast, in built-up areas, most of the pixels of GF-1710, GF-1705, and GF-1708 were located in the upper right-hand portion of the map, similar with those of A2-1504, A2-1601, and R2-1607, showing medium entropy multiple scattering. ALOS-2 data (Figure 7e–g) presented good consistencies in built-up areas over Beijing, but GF-3 data (Figure 7a–c) displayed some differences, such as lower entropy values for (a) GF-1703 and lower alpha values for (c) GF-1712. Providing that GF-1703 contained more CTs, the decreased entropy was explicable [19]. For vegetation, GF-3 had similar pixels with RADARSAT-2 and ALOS-2 distributed in medium and high entropy portions adjacent to the curve, representing the bound of the minimum observable $\bar{\alpha }$ value as a function of entropy. GF-1702 was an exception, where most pixels were concentrated in the medium entropy portion. For water, GF-3 also had similar pixels to RADARSAT-2, distributed in the medium right-hand portion of the *H–*$\bar{\alpha }$ map. The high entropy of water can be explained as water had a low backscattering power for both C-band or L-band sensors and, thus, the observed coherency matrix of water had approximate low eigenvalues. Also, the waves in water surfaces can increase the randomness of scattering. In general, high entropy means that there is a high scattering order or random scattering with approximate eigenvalues [29]. However, the pixels in water of ALOS-2 exhibited some differences between Beijing and Wuhan.

**Figure 8:**
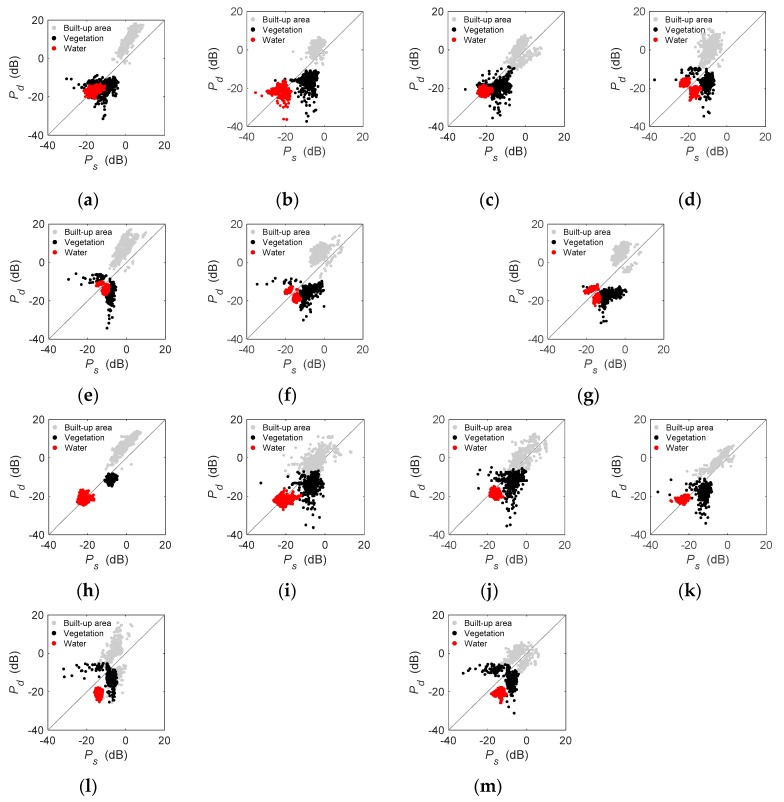
Scatter diagrams of built-up areas, vegetation, and water in double-bounce and surface-scattering powers. (**a**) GF-1703, (**b**) GF-1710, (**c**) GF-1712, (**d**) R2-0903, (**e**) A2-1603, (**f**) A2-1610, (**g**) A2-1612, (**h**) GF-1702, (**i**) GF-1705, (**j**) GF-1708, (**k**) R2-1607, (**l**) A2-1504, and (**m**) A2-1601.

As shown in Figure 8, both *P_s_* and *P_d_* showed much higher values for built-up areas than vegetation and water. Further, most of the pixels in built-up areas had larger double-bounce scattering powers than surface scattering, except GF-1712 with a lower incidence angle. However, the distributions of pixels in vegetation and water were variable for 13 images. Most pixels in GF-3 data (Figure 8a–c,h–f) displayed dominant surface scattering over forest areas, especially for (b) GF1710 and (h) GF-1702, where over 90% of pixels presented higher surface scattering power than double-bounce scattering power. By contrast, a large proportion of pixels with dominant double-bounce scattering appeared in ALOS-2 images (Figure 8e,l,m) over forest areas, especially for (m) A2-1601. This meant that the variation between GF-3 and ALOS-2 achieved expected results corresponding to the increased ability at longer wavelengths to penetrate vegetation canopies. In Beijing (Figure 8a–g), only GF-1710 and R2-0903 displayed good discrimination between vegetation and water, while the pixels in vegetation and water were mixed in other maps. By contrast, Figure 8h–m exhibited that vegetation and water could be separated in the *P_s_-P_d_* map because of the larger surface or double-bounce scattering power of the pixels in vegetation than that in water.

**Figure 9:**
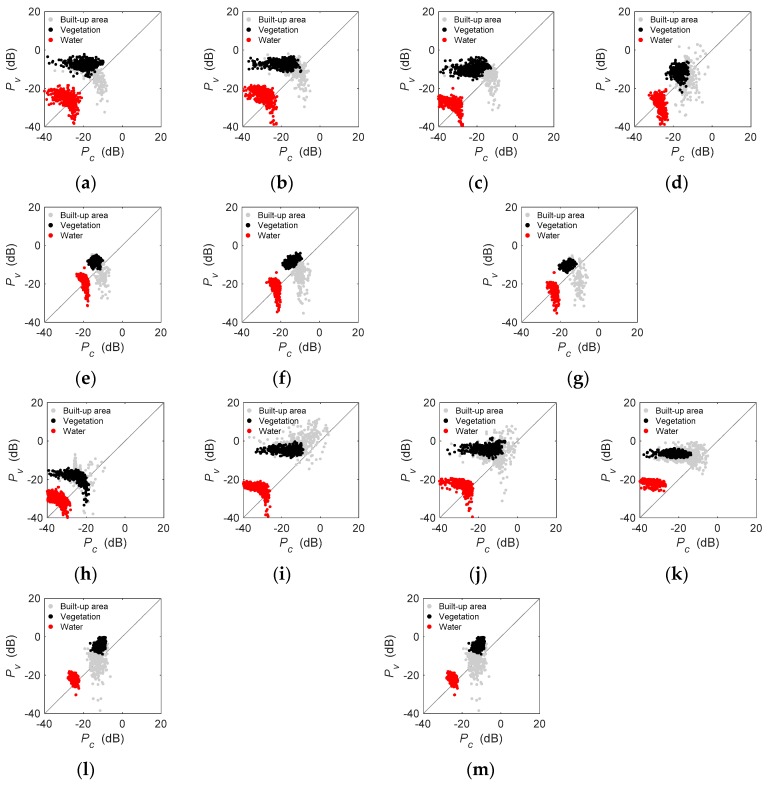
Scatter diagrams of built-up areas, vegetation, and water in volume and helix powers. (**a**) GF-1703, (**b**) GF-1710, (**c**) GF-1712, (**d**) R2-0903, (**e**) A2-1603, (**f**) A2-1610, (**g**) A2-1612, (**h**) GF-1702, (**i**) GF-1705, (**j**) GF-1708, (**k**) R2-1607, (**l**) A2-1504, and (**m**) A2-1601.

As presented in Figure 9, GF-3 and RADARSAT-2 shared similar *P_c_-P_v_* diagrams, where most of the pixels in built-up areas and vegetation were mixed, but they had much higher volumes and helix scattering powers than that in water. Compared with GF-3 and RADARSAT-2, most of the pixels in ALOS-2 obtained better helix scattering power in vegetation and water. In general, the pixel-based analysis indicated that GF-3 had similar polarimetric decomposition results with that of ALOS-2 and RADARSAT-2, and that different types of samples were significantly separated for all three sensors.

#### 3.2.4. Comparison of Classification Results

A comparison of classification results in Beijing among GF-3, ALOS-2, and RADARSAT-2 are summarized in Table 4. GF-1703, R2-0903, and A2-1603 had similar performances in land-cover classification. They obtained lower overall classification accuracies (CAs, <80%) and lower overall Kappa coefficients (KC, <0.70), as well as lower product accuracies (PA, <80%) in built-up areas and vegetation areas. GF-1712 and A2-1612 performed better, with about 83% CA and 0.70 KC. GF-1710 achieved the best performance with 91% CA and 0.83 KC. In general, the results of classification were good for GF-3 data, except the water in GF-1712 (PA, <40%; UA, <30%). For GF-1712 obtained in winter, the water frozen into ice changed the backscattering power and scattering mechanism that lead to the mixture of water and other land-cover types. Moreover, the accuracy of water was easily impacted and changed as there was only a small proportion of water in the study area over Beijing.

**Figure 10:**
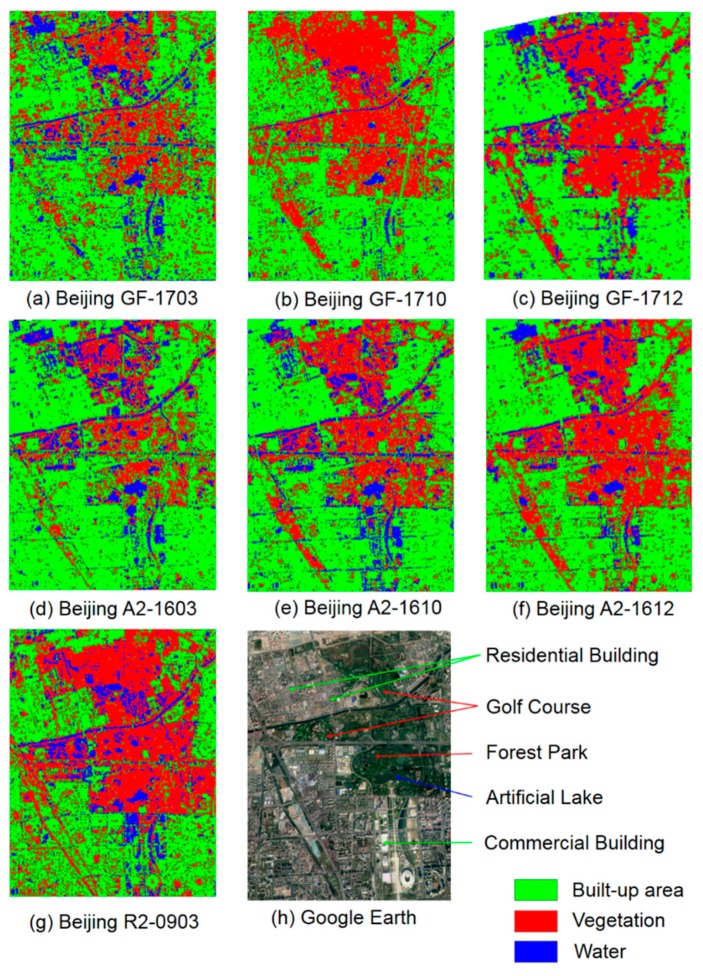
Classification of built-up areas, vegetation, and water in Beijing.

As shown in Figure 10, variable classification results of GF-3 were obtained. In GF-1703, some pixels in residential buildings were incorrectly assigned to vegetation, which also happened to R2-0903 and A2-1603. In GF-1712, some trees in forest parks, grasslands in golf courses, and commercial buildings and the ground were incorrectly assigned to water, which was also presented in R2-0903, A2-1603, and AL1610. GF-1710 performed the best, where the artificial lake and forest park were almost perfectly detected from built-up areas.

A comparison of classification results in Wuhan among GF-3, ALOS-2, and RADARSAT-2 are summarized in Table 5. GF-3 and ALOS-2 were stable and similar, around 87% CA and 0.80 KC. By contrast RADARSAT-2 performed better with 92% OCA and 0.89 OKC. All three GF-3 images acquired lower product accuracies (PA < 75%) in built-up areas.

**Figure 11:**
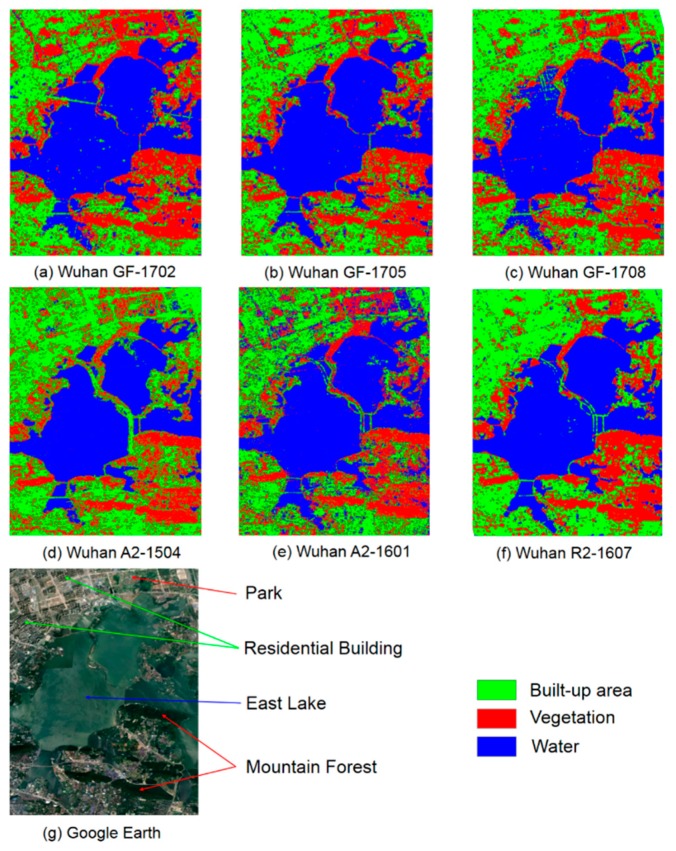
Classification of built-up areas, vegetation, and water in Wuhan.

As shown in Figure 11, the classification results of GF-3 were found to be stable. However, GF-1702 demonstrated that some pixels in residential buildings under construction were assigned to vegetation. In contrast, RADARSAT-2 had a better performance in built-up areas, while ALOS-2 incorrectly assigned some pixels in built-up areas and vegetation to water. For all classification results, with the changes of the image-acquiring situations, the classification accuracy of GF-3 experienced a variation from 75.0% to 91.4%, similar to that of ALOS-2 and RADARSAT-2.

## 4. Discussion

### 4.1. Difference between C-Band and L-Band

Differences exist in the performance of individual C-band or L-band data in its application [33,37,38,39]. Given the increased ability at longer wavelengths to penetrate vegetation canopies, the pixels in vegetation should be more concentrated at high entropy for the C-band data because of the predominating canopy volume-scattering mechanisms [25]. Table 3 indicated that C-band PolSAR images (GF-1703 and R2-0903) outperformed the L-band image (A2-1603) regarding similarity of backscatter coefficients. Also, as shown in Figure 8, the pixels in vegetation of GF-3 had a similar performance to RADARSAT-2, but different from ALOS-2. In addition, the classification results indicated that vegetation in Wuhan obtained a generally higher product accuracy than that in Beijing (Table 4 and Table 5). Since effective surface roughness of a scattering boundary is relative to the wavelength of the incident microwaves, there may be a difference in backscatter levels of C-band and L-band data [40]. Nevertheless, the discrimination between the pixels of built-up areas, vegetation, and water of GF-3 and ALOS-2 was generally similar in *Pauli_B_ + Pauli_C_ − Pauli_A_*, *H*–$\bar{\alpha }$, *P_s_-P_d_,* and *P_v_-P_c_* maps.

### 4.2. Incidence Angle Effects

The use of images acquired from different incidence angles sometimes leads to undesired variations in performance [41]. Among the 13 used images, incidence angles changed from 20° to 45°. For GF-3, GF-1712 possessed the lowest incidence angle (< 22°); GF-1702, GF-1705, GF-1708, and GF-1710 had medium angles (around 37°) similar to A2-1504, A2-1601, A2-1604, and R2-0903; and GF-1703 had the highest incidence angle (> 45°) similar to R2-1607. As shown in Figure 7c, the pixels in built-up areas with lower incidence angles obtained lower $\bar{\alpha }$ than other images, and presented a dominant dipole-type scattering mechanism, rather than a double-bounce type. Also, as presented in Figure 8c, the pixels in built-up area did not clearly perform expected double-bounce scattering phenomena. The pixels in GF-3 with higher incidence angles had similar performances as others.

### 4.3. Seasonal Effects

The images used were acquired in different seasons that may lead to variations in the classification results [42]. In Beijing, GF-1710, acquired in the mid-autumn before trees began to shed their leaves, was found to have the best classification results (91.4% OCA, 0.837 OKC). In contrast, GF-1703 and GF-1712 acquired in winter before trees became green achieved lower product accuracies and user accuracies of vegetation, similar with A2-1603 and A2-1612 (Table 4). In Wuhan, little impact from the season on the product accuracy and user accuracy of vegetation was observed (Table 5), resulting from the fact that most of the mountain trees in Wuhan were evergreen.

### 4.4. Difference between Beijing and Wuhan

The difference in land-cover structure between Beijing and Wuhan give rise to the varied histograms of the backscattering coefficients in each polarimetric channel. As almost half of the study area in Wuhan was water, all of the histograms in Wuhan presented double peaks because water has a distinctly lower backscattering power than other land-cover types (Figure 5 and Figure 6). Because the histograms in Beijing just have a single peak, they had higher similarities between GF-3 and RADARSAT-2 with a shorter Bhattacharyya distance than that in Wuhan (Table 3). In addition, the building samples in urban areas of Beijing and Wuhan exhibited different characteristics, such as size, shape, orientation, and space interval. Nevertheless, most of the pixels in built-up areas of Beijing and Wuhan both presented a larger double-bounce scattering power than surface scattering for all three sensors. The classification results also displayed similar and lower product accuracy in built-up areas of both Beijing and Wuhan (Table 4 and Table 5). Due to the mixture and coexistence of built-up structures, vegetation water areas and the heterogeneity of the objects (e.g., residential with gardens) resulted in different backscattering variations within these areas of homogenous land-cover classes [24]. Despite the difference in the vegetation species in Beijing and Wuhan, the two sample areas had similar distribution characteristics in the pixel-based analysis. However, the product accuracy of vegetation in Wuhan was found generally higher than that in Beijing for all three sensors (Table 4 and Table 5). The pixel-based analysis of water in GF-3 and RADARSAT-2 obtained similar results between Wuhan and Beijing. However, water in ALOS-2 presented equal surface and double–bounce scattering power in Beijing, while it performed larger surface scattering power than double-bounce in Wuhan (Figure 6 and Figure 8). For all three sensors, the product and user accuracy of water in Beijing was lower than that in Wuhan.

### 4.5. Polarimetric Distortion

Considering the impact of operational bands, incidence angles, and image acquiring situations, the results of the histogram-based, pixel-based, and classification analyses indirectly reflected the polarimetric fidelity of PolSAR data. In general, the histogram-based and classification analyses did not show any signal of distortion impacting GF-3 data. However, the pixel-based analysis indicated that GF-1702 and GF-1703 might suffer polarimetric distortion. Hence, it impacted the decomposition result of lower performance of polarimetric entropy as well as strange model-based decomposition results compared with other images (Figure 7, Figure 8 and Figure 9). However, the remaining four GF-3 PolSAR images obtained later had no signs showing that any polarimetric distortion imposed significant impacts on the decomposition results. It may be inferred that the sensor of GF-3 operated unsteadily before May 2017 and has been improved. Overall, the experimental results based on a three-hierarchy framework indicated that polarimetric distortions of most GF-3 PolSAR images were similar to ALOS-2 and RADARSAT-2. ALOS-2 and RADARSAT-2 have been widely applied in Earth observations for many years, and their quality is confirmed to meet the users’ requirements [11,12,15,33,37,43,44,45]. The crosstalk accuracy of RADARSAT-2 of −30 dB, the channel imbalance of 0.5 dB in amplitude, and 5 degrees in phase are reported [46]. The accuracy requirement of ALOS-2 is −30 dB in crosstalk, 0.4 dB in channel imbalance amplitude, and 5 degrees in the channel imbalance phase [6]. Generally, previous studies have documented that GF-3 has a similar polarimetric performance to RADARSAT-2 and ALOS-2 using scattering properties and corner reflectors [3,18]. In this study, the polarimetric fidelity of GF-3 PolSAR data was proved at a similar level with that of RADARSAT-2 and ALOS-2, e.g., CTs <−30 dB and CIs <0.5 dB.

## 5. Conclusions

In this paper, an innovative, three-hierarchy strategy was proposed to evaluate PolSAR data quality based on the images themselves and their applications, with the support of validation information acquired from ground infrastructure. Its evaluation ability of polarimetric performance was demonstrated by GF-3 experiments using RADARSAT-2 and ALOS-2 as references. The experiments indicated that most of the calibrated GF-3 PolSAR data remained as insignificant polarimetric distortions. However, the performance of GF-3 data obtained before May 2017 showed some differences compared to data obtained after May 2017. This suggests that the system of GF-3 may have been improved around May 2017. Moreover, the results of the present study also proved that the backscattering properties of the target could be reasonably interpreted by decomposition theory using PolSAR images of GF-3; similar performances with that of RADARSAT-2 and ALOS-2 were found. Further, the polarization information of targets included in the pixels of GF-3 is applicable to detecting and distinguishing different land-cover types. Similar abilities of GF-3, ALOS-2, and RADARSAT-2 in land-cover classifications were also found. However, considering the image acquiring situations, incidence angles, operating bands, and many other factors, GF-3 had variable results in the pixel-based analysis and classification, as well as RADARSAT-2 and ALOS-2. Hence, when using PolSAR images in a specific study, the specifications of the data should be cautiously considered to ensure appropriateness.

## Figures and Tables

**Table 1:** Specifications of the PolSAR images used.

	Imaging Time	Abbreviation	Incidence Angle (Deg)	Operating Band
**Beijing**				
GF-3	8 March 2017	GF-1703	46~47	C
GF-3	2 October 2017	GF-1710	36~38	C
GF-3	9 December 2017	GF-1712	19~22	C
ALOS-2	8 March 2016	A2-1603	38~39	L
ALOS-2	27 October 2016	A2-1610	26~28	L
ALOS-2	22 December 2016	A2-1612	26~28	L
RADARSAR-2	8 March 2009	R2-0903	39~40	C
**Wuhan**				
GF-3	12 February 2017	GF-1702	35~37	C
GF-3	29 May 2017	GF-1705	35~37	C
GF-3	24 August 2017	GF-1708	35~37	C
ALOS-2	3 April 2015	A2-1504	35~37	L
ALOS-2	8 January 2016	A2-1601	35~37	L
RADARSAR-2	6 July 2016	R2-1607	45~46	C

**Table 2:** The proportion of pixels with differences between $\sigma_{HV}^{o}$ and $\sigma_{VH}^{o}$ lower than specific values.

	Percentage (%)				
	<1 dB	<2 dB	<3 dB	<5 dB	<10 dB
GF-3	13.7	27.2	40.5	61.3	87.6
ALOS-2	15.8	32.1	44.6	65.7	87.1
RADARSAT-2	30.6	50.1	63.0	79.2	93.5

**Table 3:** Similarity of backscattering coefficients for GF-3, ALOS-2, and RADARSAT-2.

	Bhattacharyya Distance (*Bd*)		
	$\sigma_{HH}^{o}$	$\sigma_{VV}^{o}$	$\sigma_{HV}^{o}$
**Beijing**			
GF-1710 & A2-1603	0.16	0.43	0.21
GF-1710 & R2-0903	0.02	0.04	0.10
R2-0903 & A2-1603	0.11	0.41	0.14
**Wuhan**			
GF-1710 & A2-1603	0.10	0.31	0.15
GF-1710 & R2-0903	0.56	0.46	0.49
R2-0903 & A2-1603	0.69	0.10	0.48

**Table 4:** Comparison of land-cover classification results in Beijing using images from GF-3, ALOS-2, and RADARSAT-2 data.

**Land-Cover Type**	**GF-3**						**RADARSAT-2**	
	GF-1703		GF-1710		GF-1712		R2-0903	
	PA	UA	PA	UA	PA	UA	PA	UA
Built-up area	77.8	81.9	89.3	92.4	84.7	96.5	77.0	98.6
Vegetation	70.9	69.4	93.2	90.7	90.9	82.1	75.1	68.3
Water	76.1	62.8	95.3	87.9	38.1	27.5	97.3	51.7
Classification accuracy (CA)	75.0		91.4		83.1		78.3	
Kappa coefficient (KC)	0.565		0.837		0.705		0.644	
	**ALOS-2**							
	A2-1603		A2-1610		A2-1612			
	PA	UA	PA	UA	PA	UA		
Built-up area	79.2	81.0	83.4	90.1	80.6	96.9		
Vegetation	62.2	75.0	73.6	86.1	85.7	73.9		
Water	95.4	56.9	98.0	53.6	92.8	53.1		
Classification accuracy (CA)	75.1		80.4		83.1			
Kappa coefficient (KC)	0.574		0.656		0.700			

**Table 5:** Comparison of land-cover classification results in Wuhan using images from GF-3, ALOS-2, and RADARSAT-2 data.

**Land-Cover Type**	**GF-3**						**RADARSAT-2**	
	GF-1702		GF-1705		GF-1708		R2-1607	
	PA	UA	PA	UA	PA	UA	PA	UA
Built-up area	68.2	93.0	73.8	91.1	74.6	88.5	82.6	96.2
Vegetation	96.0	75.6	93.4	79.1	95.6	81.8	97.8	68.3
Water	98.4	96.9	98.7	98.1	95.8	95.5	98.4	98.0
Classification accuracy (CA)	87.1		88.7		88.3		92.7	
Kappa coefficient (KC)	0.807		0.831		0.824		0.890	
	**ALOS-2**							
	A2-1504		A2-1601					
	PA	UA	PA	UA				
Built-up area	81.9	84.5	74.1	93.5				
Vegetation	85.9	86.3	84.0	84.1				
Water	98.2	95.0	98.9	83.7				
Classification accuracy (CA)	88.8		86.3					
Kappa coefficient (KC)	0.831		0.793					
